# Characterization of novel pesti- and astro-like viruses in hatchery-reared European seabass (*Dicentrarchus labrax*) associated with health disorders and low mortality

**DOI:** 10.1186/s13567-026-01758-2

**Published:** 2026-05-19

**Authors:** Lénaïg Louboutin, Joelle Cabon, Pauline Grippon, Veronique Beven, Edouard Hirchaud, Matthieu Jamin, Pierre-Marie Boitard, Yannick Blanchard, Thierry Morin

**Affiliations:** 1 ANSES, Agence Nationale de Sécurité Sanitaire de L’Alimentation, de L’Environnement Et du Travail-Laboratoire de Ploufragan-Plouzané-Niort, Unité Virologie, Immunologie Et Écotoxicologie Des Poissons, INR Emerg’In, Plouzané, France; 2 ANSES, Agence Nationale de Sécurité Sanitaire de L’Alimentation, de L’Environnement Et du Travail-Laboratoire de Ploufragan-Plouzané-Niort, Unité Virologie Porcine, Innovation Et Génomique, Ploufragan, France; 3 Filiavet Réseau Cristal, 15 Rue du Puits, Saint-Martin-Des-Champs, France

**Keywords:** Virus, seabass, pestivirus, astrovirus, pathogenicity

## Abstract

European seabass is a major farmed species in Mediterranean aquaculture. Its early life stages are very sensitive to sanitary and environmental conditions and are therefore closely monitored. In this study, we characterized new viruses recently isolated from fingerlings in a hatchery showing whitish three-dimensional lesions on the fins and sides and distended abdomens and investigated their pathologic potential. A cytopathic effect was observed 17 days post-inoculation (dpi) of homogenized internal organs at 20 °C on a CHSE_214_ cell line. Viral presence was confirmed using the indirect fluorescent antibody test (IFAT) targeting double-stranded RNA patterns. Next-generation sequencing (NGS) and phylogenetic analyses were used to characterize two viruses: a pesti-like virus of 13,123 nt detected both in positive supernatant and organ homogenate, named *Dicentrarchus labrax* Pesti-like virus (DLPLV); and an astro-like virus with a sequence of 5,068 nt detected only in the homogenate and clustering with other described fish astroviruses, and named *Dicentrarchus labrax* Astro-like virus (DLALV). In vivo, direct or waterborne contact of healthy fish with fish showing clinical signs induced very sporadic and fleeting clinical signs, while bath infection with DLPLV alone induced 8 to 16% mortality for 162-days post-hatching fish. Seroneutralization tests demonstrated the existence of a humoral response specific to DLPLV, regardless of the route of infection. This work highlights the wide diversity of aquatic viruses and illustrates the complexity of reproducing experimentally the clinical signs observed in hatcheries, especially in the case of multifactorial phenomena. The diagnostic tools developed will be usable for surveillance and fast detection of any new clinical cases.

**Supplementary Information:**

The online version contains supplementary material available at 10.1186/s13567-026-01758-2.

## Introduction

One of the main challenges of the coming decades will be feeding nine billion people in the context of global climate change, which has been recognized as a fundamental threat to the security of the food supply and consequently, sustainable development, since the Paris Agreement in 2017 (COP21). Aquaculture production in 2022 (94.4 million tons) represented 51% of the total world production of aquatic species and occupies a strategic place in producing high quality food both in terms of nutritional quality and safety, but also to ensure economically and socially sustainable development [1]. Importantly, the contribution of aquaculture to the supply of aquatic resources intended for human consumption is expected to increase in the future in response to the depletion of wild stocks. Nevertheless, rearing fish at stressful high densities and, in most cases, in open environments that are difficult to control creates ideal conditions for the emergence of infectious diseases, even when biosecurity measures are implemented. In addition to the large number of existing farming species and ecosystem diversity, this can explain the dynamics of the emergence of aquatic viral pathogens observed in recent decades [2, 3].

Cell culture remains an efficient non-specific method for detecting viruses in aquatic organisms showing atypical clinical symptoms, with the advantage of amplifying the unknown virus, simplifying subsequent analyses, and giving the possibility to demonstrate Koch’s postulate. The recent development of high-throughput sequencing is a complementary tool of high value for the deeper characterization of viral metagenomes associated with organisms. When an unknown virus cannot be amplified in vitro, unbiased high-throughput sequencing can be performed directly on tissue samples [3–5]. The most recently characterized emerging viral pathogens using these tools include: Tilapia lake virus, identified as the etiological cause of considerable mortalities of tilapia in Israel and Ecuador in 2009 [4]; Piscine reovirus (PRV) fully sequenced and associated with heart and skeletal muscle inflammation syndrome (HSMI) after experimental infection of salmon and analysis performed on heart and serum [6]; and Shrimp hemocyte iridescent virus isolated from *Litopenaeus vannamei* in China in 2017 [7]. A toti-like virus was also isolated and genetically characterized from farmed seabass larvae displaying nonspecific clinical signs [8].

The family *Flaviviridae* comprises small enveloped, positive-sense, single-stranded RNA viruses belonging to four genera: *Flavivirus*, *Pestivirus*, *Hepacivirus* and *Pegivirus* [9, 10]. Although mammals and birds are known to be classical hosts, recent metagenomic analyses of aquatic organisms have identified several *Flavivirus* genus members [11]. The first description of a member of the *Flaviviridae* family in fish was reported recently, in 2018, in a Norwegian lumpfish (*Cyclopterus lumpus*) hatchery, where mortality of at least 50 percent was observed [12]. The Wenzhou shark flavivirus was later identified in the Pacific spadenose shark (*Scoliodon macrorhynchos*). This virus was also found in the gazami crab (*Portunus trituberculatus*) in a study describing several novel flaviviruses that infect crustaceans and cephalopods [13, 14]. More recently, a salmon flavivirus was sequenced from moribund Chinook salmon (*Oncorhynchus tshawytscha*) from the Eel River in California, USA [15]. Pestiviruses have been isolated only from pigs or ruminants (cattle, goats and sheep). A recent metagenomics-based study identified a pestivirus in Glass knifefish (*Eigenmannia virescens*), expanding the pestivirus host range to a wide diversity of vertebrates, including lower vertebrates [16]. Nevertheless, the full host range and effects of these aquatic flaviviruses remain poorly described. Their genome, which is approximately 12,300 nucleotides long, is composed of a single open reading frame that encodes a polyprotein precursor of 3900 amino acids. In 2017, Smith et al*.* proposed a taxonomic classification of the genus *Pestivirus* introducing seven new species in addition to the four previously recognized ones [17]. This classification was modified again in 2021, with the introduction of eight new species, distinct from the former members, named *Pestivirus* L to S, in addition to the 11 genera, codified as *Pestivirus* A to K [18]. Recently, the first genome-wide multiple sequence alignment (MSA) of all known pestivirus species was proposed [19]. A distinctive feature of this genus is the E^rns^ glycoprotein envelope, which has RNase activity, and a non-coding protease N^pro^. Those two proteins disrupt host antiviral mechanisms [20–22].

The family *Astroviridae* comprises small, non-enveloped, positive-sense single-stranded RNA viruses with a genome of approximately 6–8 kb in length. They can infect a wide variety of terrestrial mammals and are typically associated with gastrointestinal illness [23]. Although only two genera, *Avastrovirus* and *Mamastrovirus*, have been recognized by the International Committee on Taxonomy of Viruses (ICTV) for avian and mammalian astroviruses, respectively, in the last decade, many astroviruses and astrovirus-like genomes have been identified in various other vertebrates and invertebrates [24]. Shi et al*.* [13] recently identified astrovirus sequences in several fish species using a large-scale meta-transcriptomic approach; however, their origin and potential impact are still unclear [13, 25].

The European seabass (*Dicentrarchus labrax*) is the most widespread marine fish species in the Mediterranean Sea, the Black Sea and the northeastern Atlantic. It was also one of the first marine species to be cultured in Europe, mainly in Greece and Spain [26, 27]. Here, we report the characterization of a putative pestivirus and an astrovirus from fingerlings showing health disorders and low mortality in a European seabass hatchery. The phylogenetic positions of these two putative viruses were analyzed after full-length sequencing, as well as their capacities to reproduce Koch’s postulate.

## Materials and methods

### Samples for diagnosis

Seabass fingerlings (4-cm long) raised at a water temperature of 18 °C and showing external clinical signs were sampled from a hatchery at two different times, in June 2016 (sample identification OO76) and September 2016 (16/219). Samples were stored at a temperature below 10 °C until analysis.

### Bacteriological and histological investigations

Moribund fish were euthanized for necropsy on site (n = 5). A general examination was performed with skin scraping, gill sampling for microscopical examination, and coelomic exam including macroscopical lesions inventory and direct bacterioscopy on spleen, kidney and ascites. Blood was collected from the kidney during the necropsy and inoculated on trypticase soy agar + 1.5% NaCl at 22 °C for 96 h. Fragments of skin, muscle, brain, gills, heart, stomach, intestine, spleen, liver and kidney were fixed in 10% formalin for histological examination. In the case of bacterial growth, bacterial identification of the different colony morphotypes was carried out using matrix-assisted laser desorption/ionization–time of flight MALDI-TOF (service provided by Labocéa, Quimper, France).

### Cell culture isolation and characterization

For each sample, OO76 and 16/219, approximately 1 g of internal organs (kidney, spleen, heart, brain and eyes) from 10 fingerlings was homogenized in a mortar and pestles with sand and placed in 9 mL of Eagle’s solution containing antibiotics (200 IU.mL^−1^ penicillin G, 0.2 mg.mL^−1^ streptomycin, and 0.2 mg.mL^−1^ kanamycin). Ascites fluid was also collected for sample OO76, and tissues from whitish three-dimensional lesions were obtained for sample 16/219. The samples were centrifuged (2,000 × *g*) for 15 min at 5 ± 3 °C. Then, 100 µL of the supernatant were inoculated onto 24-well plate cultures for a total number of five cell lines: bluegill fry (BF-2, [28]), epithelioma papulosum cyprini (EPC, [29]), Chinook salmon embryo (CHSE_214_, [30]), sea bass larvae (SBL, [31]) and rainbow trout gonad (RTG-2, [32]). The cell cultures were incubated at 20 ± 2 °C and checked regularly for the development of a cytopathic effect (CPE). A blind passage was performed 7 days after using the same cell lines. When a CPE was observed, the supernatant was filtered through a 0.45 µm filter, and a chloroform test was performed to check for the presence of a lipid bilayer viral envelope, as described by Louboutin et al*.* [33]. The viruses isolated from these samples were named OO76_s_ and 16/219_s_.

### Indirect fluorescent antibody test (IFAT)

IFAT was performed using the specific double-stranded (ds) RNA antibody J2, which enables the detection of viral RNA or DNA, as dsRNA does not exist in uninfected eukaryotic cells. CHSE_214_ cell lines plated in 4-well plates were infected with tenfold serial dilutions of OO76_s_ or 16/219_s_ (one well per plate was used as negative control). Three days post-inoculation, plates were fixed with a mixture of acetone:ethanol (150:350) for 15 min, and the IFAT protocol was carried out as described by Louboutin et al*.* [8].

### Transmission electron microscopy (TEM)

The OO76_s_ was propagated on the CHSE_214_ cell line in a 75 cm^3^ flask at 20 °C. On days 5 and 7, the medium was removed, and infected and non-infected cells were rinsed with 0.1 M pH 7.2 cacodylate buffer. The fixing buffer glutaraldehyde 2%—paraformaldehyde 2%—cacodylate buffer 0.1 M, pH 7.2 was added and the sample was left to stand for 1 h at room temperature (RT). The cells were rinsed three times with cacodylate buffer. After buffer removal, 1% osmium solution was added for 1 h at RT, and the cells were rinsed three times. The cells were then placed in contact with 0.4% uranyl acetate solution overnight at 5 ± 3 °C. Dehydration was performed using successive ethanol baths (from 50 to 100 °C). Scraped cells were collected in a microtube before centrifugation, and the pellet was eventually included in the resin. Imaging was performed using a JEM-1400 electron microscope (JEOL Ltd., Tokyo, Japan) at the TEM facility, University of Brest, France.

### Nucleic acid extraction, library preparation, and high-throughput sequencing

RNA was extracted either from the supernatant of a CPE-positive 0076_s_ sample or from a three-dimensional lesion homogenate prepared from diseased fingerlings 16/219 (subsequently named 16/219_p_) with Trizol–LS, according to the manufacturer’s instructions. Library preparation followed, after rRNA depletion with a RiboMinus™ Low-Input RNA-Seq Kit (Ambion, Austin, TX, USA), as described by the manufacturer. The RNA library was obtained using an Ion Total RNA-Seq Kit v.2 (Life Technologies, Carlsbad, CA, USA), according to the manufacturer’s recommendations. The libraries were sequenced using an Ion Proton™ Sequencer and an Ion PI™ Chip v.2 (Life Technologies). The reads were cleaned with Trimmomatic 0.36 software (ILLUMINACLIP: oligos.fasta: 2:30:5:1: true; LEADING: 3; TRAILING: 3; MAXINFO: 40:0.2; MINLEN: 36), and alignment using Burrows–Wheeler Aligner (BWA) (v.0.7.8) was performed using cleaned reads on the Chinook salmon genome. All reads that did not align with the Chinook salmon genome were collected and submitted for assembly using the Mira de novo assembler (v.4.0.2.) Mira contigs were then aligned on a local nt database with blastn v.2.4.0 for identification. Blastn unidentified sequences were manually curated for identification with a BlastX homology search or open reading frame search with ORFFinder. Viral genome structures and sequences were validated and corrected by mapping all reads and assembled contigs against the newly identified viral genomes as references using BWA, and read alignment was visualized using Integrative Genomics Viewer (IGV).

### Phylogenetic analyses

Phylogenetic analyses were performed according to Shi et al*.* [13], supplemented with *Flaviviridae* member virus sequences downloaded from Mifsud et al*.* [16]. The first step was multiple sequence alignment performed using MAFFT (Multiple Alignment using Fast Fourier Transform, version 7) of our sequence and the genomes used by Mifsud et al*.* on the NS5B amino acid sequence. To optimize the phylogenetic tree, multiple sequence alignment cleaning was performed using TrimAl to retain only the regions relevant to phylogeny. Phylogenetic reconstructions were generated on 428 sequences of 172 amino acids of Flaviviridae family members using the maximum likelihood (ML) method, after selecting the best-fit model with IQ-TREE 2.3.5 built July 10, 2024 [34, 35] and using ultrafast bootstrap approximation [36]. A second phylogenetic analysis based solely on pestivirus genomes (45 polyprotein GenBank references) was performed according to Postel et al*.* and Mifsud et al*.* [16, 18, 37]. A partial alignment of 314 amino acids corresponding to partial NS5B protein was generated by Geneious (Geneious Prime 2023.2.1 built July 20, 2023). After cleaning with TrimAl, an ML phylogenetic tree was obtained with IQ-TREE and the model LG + I + R4. Phylogenetic trees were represented and annotated using iTOL [38].

Regarding astro-like virus, RdRp amino acid sequences were selected and filtered to keep only sequences longer than 150 residues. Multiple sequence alignment using MAFFT was performed with 40 sequences, including the new European seabass virus. The alignment was trimmed (319 residues), and an ML tree was built using the LG + R5 model and IQ-TREE, as described previously.

### PCR tools for detection and quantification

A primer pair oPVP469 5′-ATGAAAACGGAGCAGAGGCA-3′ and oPVP470 5′- TGGATCGTCAAGGGGGTTTG-3′. was designed (Primer3plus) and located at positions 10651 to 11354 of the putative pestivirus genome. OO76_s_ RNA was extracted from 200 µL of positive cell culture supernatant using a NucleoSpin^®^ Virus Kit (Macherey–Nagel, GmbH & Co. KG, Düren, Germany). The 704-base pair (bp) fragment was amplified via a one-step conventional RT-PCR (RT-cPCR) using the SuperScript^™^ III One-Step RT-PCR System with Platinum™ Taq High Fidelity DNA Polymerase (Invitrogen, Carlsbad, CA, USA). The reaction contained approximately 1 µg of RNA, 400 nM of each primer, 1 µL of Taq, 25 µL of reaction mix, and nuclease-free water to a final volume of 50 µL. RT-cPCR was carried out in a T100™ Thermal Cycler (Bio-Rad Laboratories, Hercules, CA, USA) with reverse transcription at 52 °C for 30 min, initial denaturation at 94 °C for 2 min, followed by 40 cycles of 94 °C for 15 s, 62 °C for 30 s and 68 °C for 60 s. The expected fragment size was confirmed by electrophoresis on a 2% agarose gel. Selected samples were subsequently subjected to Sanger sequencing to verify consistency between the sequences obtained by NGS and the amplified fragments. PCR products obtained after amplification of nucleic acids were purified using a NucleoSpin^®^ Gel and PCR Clean-up Kit (Macherey–Nagel) and cloned using a TOPO TA Cloning™ Kit (Invitrogen). Three clones were selected and sequenced in both directions, and all nucleotide differences were visually checked using VectorNTI v.11.5 software. An additional set of primers (oPVP645 5′-GCCATTCCAAAGAACGAAAA-3′, and antisense primer oPVP646 5′-CCATGCCAGTCTGAATGATG-3′) was used to amplify a 117-bp long fragment by quantitative SYBR green RT-qPCR. An RT-qPCR assay was also designed for the putative astrovirus, targeting an 88-bp long fragment with the following primers: oPVP743 5′-AAAAGGCGGGCCAGTACTAG-3′ and oPVP742 5′-TCGCGTTTCTCTCACTGCTT-3′. For these two RT-qPCRs, reverse transcription and amplification were performed with QuantiFast SYBR Green RT-PCR (QIAGEN, Hilden, Germany), using the following mix: 5 µL of extracted RNA added to 1 µM of each primer, 0.25 μL of QuantiFast RT mix, 12.5 μL of reaction mix (2X), and nuclease-free water in a final volume of 25 μL. RT-qPCRs were conducted in a QuantStudio^™^ 5 Real-Time PCR System (Applied Biosystems, Foster City, CA, USA), with an initial step at 50 °C for 10 min, followed by one step at 95 °C for 5 min, then 40 cycles at 95 °C for 10 s and 60 °C for 30 s. The results were expressed as cycle threshold (Ct). At the end of the program, melting curve analysis was performed at temperatures ranging from 65 °C to 95 °C.

### Experimental trials

#### Fish

A total of 445 seabass fingerlings, 345 at 162 days post-hatching (dph) with a mean weight of 2.3 g and 100 younger fish aged 51 dph, mean weight 0.2 g, were used. Before the experiments, some individuals from each batch underwent virological analyses (inoculation on EPC, BF-2 and SSN-1 cell lines) and bacteriological analyses (non-selective medium) to confirm the absence of cultivable pathogenic agents. CHSE_214_ were additionally used to check the fish status for pesti-like virus.

#### Experimental design

##### Authorization to conduct research and ethical aspects

All protocols were carried out in accordance with the European Commission Recommendation 2007/526/EC on revised guidelines for the accommodation and care of animals used for experimental and other scientific purposes. The ANSES Plouzané site is authorized to conduct experiments with live fish according to Administrative Order No. C29-212–3 issued by the Prefecture of the Finistère Department. Furthermore, the procedure was approved by the local ethics committee on animal experimentation (COMETH ANSES/ENVA/UPC No. 16) and authorized by the French Ministry for Education, Higher Education and Research under No. 04707.03.

##### Experimental system

Assays were carried out in tanks powered with filtered, UV-treated, continuously flowing (open circuit) seawater to maintain optimal water conditions for the fish throughout the experiment (oxygen saturation > 80%, pH close to 8, and water free of nitrates and nitrites). Fish were maintained under a natural light/dark cycle (14 h/10 h in winter) in a room with an air volume that changed every hour. The water temperature was regulated at 19 °C ± 2 °C and recorded with a Cobalt™ wireless probe (Oceasoft, Marseille, France) coupled with an acquisition system (ThermoClient v.4.1.0.24). Throughout the experiments, fish were fed twice a day with commercial mini-pellets NeoGrower Extra Marin (Le Gouessant Aquaculture, Lamballe-Armor, France). The effluents were treated with ozone before discharge, using a process whose virucidal efficacy has been validated on bacteriophage MS2.

#### Viral challenges

##### Effect of simultaneous infection with pesti- and astro-viruses and route of spread

A cohabitation trial was carried out in a 50 L tank. Twenty-five disease-free fingerlings at 162 dph were placed in the same tank with 15 specimens exposing distended bellies (shedders) originating from the infected stock, which was positive for both pesti- and astrovirus (viral load checked on five fish from the shedders batch: Ct_pesti=_14.9 and Ct_astro_ = 29.4). A basket containing 20 other disease-free fingerlings was added to the tank to prevent direct contact with fish showing symptoms. Mortality was monitored for 60 days post-infection (dpi). Fish were sampled (pool of skin and viscera) at 36 dpi (1 pool of 3 fish) and 58 dpi (3 pools of 3 fish) and analyzed using RT-qPCR targeting pestivirus or astrovirus.

In parallel, 40 fish (51 dph; 20 fish per experimental group) were infected by either three hours bath or intraperitoneal (IP) injection with 16/219_p_ (Ct_pesti_ = 22.9 and Ct_astro_ = 21.5; 2 mL inoculum (prepared as described in Sect. “Cell culture isolation and characterization”) in a bath of 200 mL of hyperoxygenated seawater; 50 µL injected/fish after anesthesia procedure of one minute in a 20 ppm Eugenol bath). Negative controls were included for each experimental group. Morbidity and mortality were monitored for one month, and sampling with pools of three fish per experimental group was performed at 10, 14, 21 and 31 dpi. The skin was analyzed separately from the viscera, and RT-qPCR targeting pestivirus or astrovirus were performed.

##### Virulence and infection kinetics of pestivirus

In a supplementary assay, 300 fingerlings at 162 dph, split into 6 tanks of 50 L (2 replicates per experimental group), were either bathed for 3 h in 3 L of hyperoxygenated seawater containing 1.5E + 7 50% tissue culture infectious dose (TCID_50_) of OO76_s_ (3^rd^ passage on CHSE_214_) or IP injected after Eugenol anesthesia with 50 µL of an OO76_s_ suspension at 4.75E + 5 TCID_50_.mL^−1^. For each experiment, negative controls were included, which were bathed or IP injected with equivalent volumes of an Eagle’s solution containing antibiotics instead of the OO76_s_ viral suspension. During the viral challenge, general behavior, presence of clinical signs, such as extended bellies or pustules, and mortalities were recorded daily. Dead individuals were stored at −20 °C before viral examination. At 53 dpi, some survivors (n = 3/condition) were euthanized using a lethal dose of Eugenol (100 ppm) and further analyzed (pool of skin and viscera) for viral presence. At 81 dpi, 70 fish (20 control, 40 infected by bath and 10 infected by IP injection) were anesthetized with Eugenol (20 ppm) to enable blood sampling.

To complete this assay, 40 younger fingerlings at 51 dph (20 per condition) were either infected by immersion or IP injection with OO76_s_, supernatant collected after 36 passages on CHSE_214_ cell line (Ct = 12.5; 5 mL inoculum in a bath of 200 mL; 50 µL injected/fish). Twenty additional animals were used as negative controls (bath with sterile culture supernatant). Morbidity and mortality were monitored for one month, and sampling of pools, consisting of three fish per experimental group, was performed at 10, 14, 21 and 31 dpi. The skin was analyzed separately from the viscera, and RT-qPCR targeting pestivirus was performed on the samples.

All technical details regarding the experimental trials described in this section are summarized in the Additional file 1.

#### Serum neutralization test specific to pestivirus

The serum neutralization (SN) test was performed using an endpoint technique in 96-well cell culture microplates (Nunc, Roskilde, Denmark). Sera were obtained at the end of the experimental trial performed on 162 dph fingerlings infected with OO76_s_ (81 dpi) as follows: 20 sera from non-infected fish, 40 from bath-infected fish, and 10 from IP-infected fish. The sera were heat-inactivated at 45 ± 2 °C for 30 min and then twofold serially diluted from 1/40 to 1/320 in a final volume of 52 µL of Eagle’s medium using round-bottom 96-well microplates (Nunc). An equal volume of viral suspension at a concentration between 1E + 3 and 3E + 3 TCID_50_.mL^−1^ (corresponding to a concentration between 50 and 150 TCID_50_ of virus.well^−1^) was added to each well containing the diluted sera. Plates were stirred for 5 min before being incubated overnight (approximately 18 h) at 5 ± 3 °C for the serum neutralization reaction. Control plates were prepared simultaneously, each containing wells for CHSE_214_ cell control (wells containing cell culture medium only), OO76_s_ reference virus (viral suspension used in the test, 2E + 3 TCID_50_.mL^−1^) and negative serum (without OO76 antibodies) two-fold diluted from 1/40 to 1/5120. Titration of the stock of OO76_s_ suspension used in the SN test was also included in twofold dilutions from 1/2 to 1/256. Each serum sample was analyzed in duplicate. After overnight incubation, 100 µL of the control and assay plates were transferred onto 24-h-old confluent cell monolayers, without discarding the cell culture medium. The total volume in each well was 200 µL. Plates were incubated at 20 °C for 12–15 days, fixed with formaldehyde 3.7% (Thermo Fisher Scientific, Waltham, MA, USA) for 1 h, and stained with crystal violet 0.1% (Sigma-Aldrich, St. Louis, MO, USA) for at least 1 h. A CPE was further observed using light microscopy. The results obtained from the samples were read after validation of the following controls: i) intact cell monolayers should be observed for CHSE_214_ cell control wells, while approximately 50% destruction was expected for OO76_s_ reference virus with OO76-negative serum; ii) the titer of the added OO76_s_ virus suspension should be between 50 and 150 TCID_50_.well^−1^. The neutralizing titer was defined as the inverse of the initial serum dilution, which resulted in 50% neutralization of the reference OO76_s_ virus compared to the OO76_s_ control virus and negative serum. A sample was considered positive if its antibody titer was ≥ 80.

## Results

### Clinical signs, bacteriological and histological investigations

**Figure 1:**
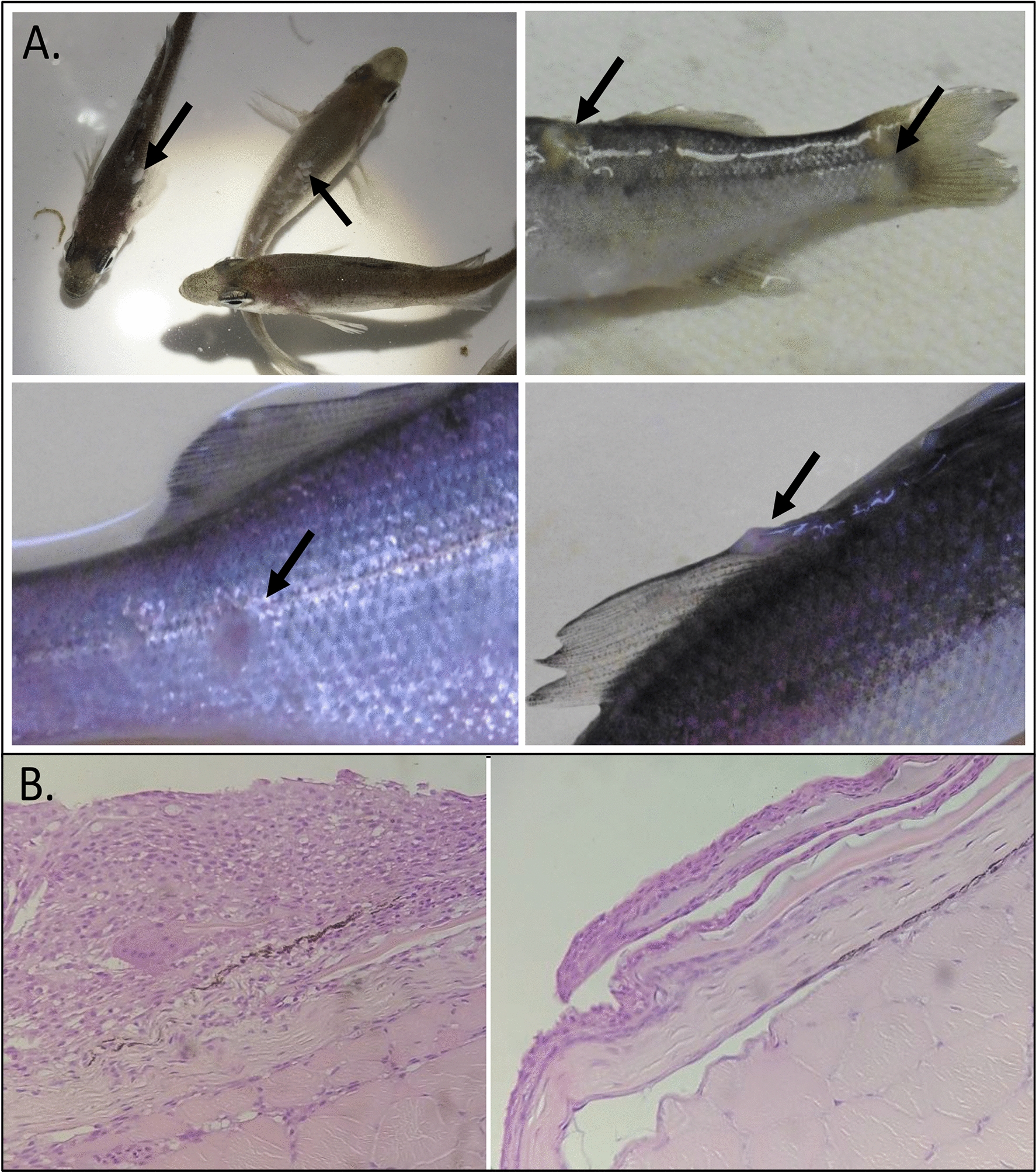
**Clinical signs observed on seabass fingerlings.** **A** Seabass fingerlings with whitish three-dimensional lesions on fins and on the back (different magnifications); **B** Hyperplastic epidermis (left) observed in fish showing clinical signs in comparison with a normal epidermis (right).

In May 2016, signs of cutaneous pathology were detected on seabass fingerlings in a hatchery. Several batches in various tanks were affected. Whitish three-dimensional lesions were observed, particularly on the fins and sides, and some fish showed distended abdomens (Figure 1A). Necropsy revealed general anemia and hepatic petechia. Histological investigations revealed hyperplastic epidermis (Figure 1B). Ascites from fish with distended bellies were found to be sterile, and feces were stringy with a fatty whitish aspect. Symptomatic fingerlings were sampled for laboratory analyses (sample OO76). Bacteriological isolation showed the presence of *Aliivibrio fischeri* in the renal tissue of 4 of the 5 samples analyzed. Some bacteria of the genus *Tenacibaculum* were also detected on the gills.

### Cell culture isolation and characterization

**Figure 2:**
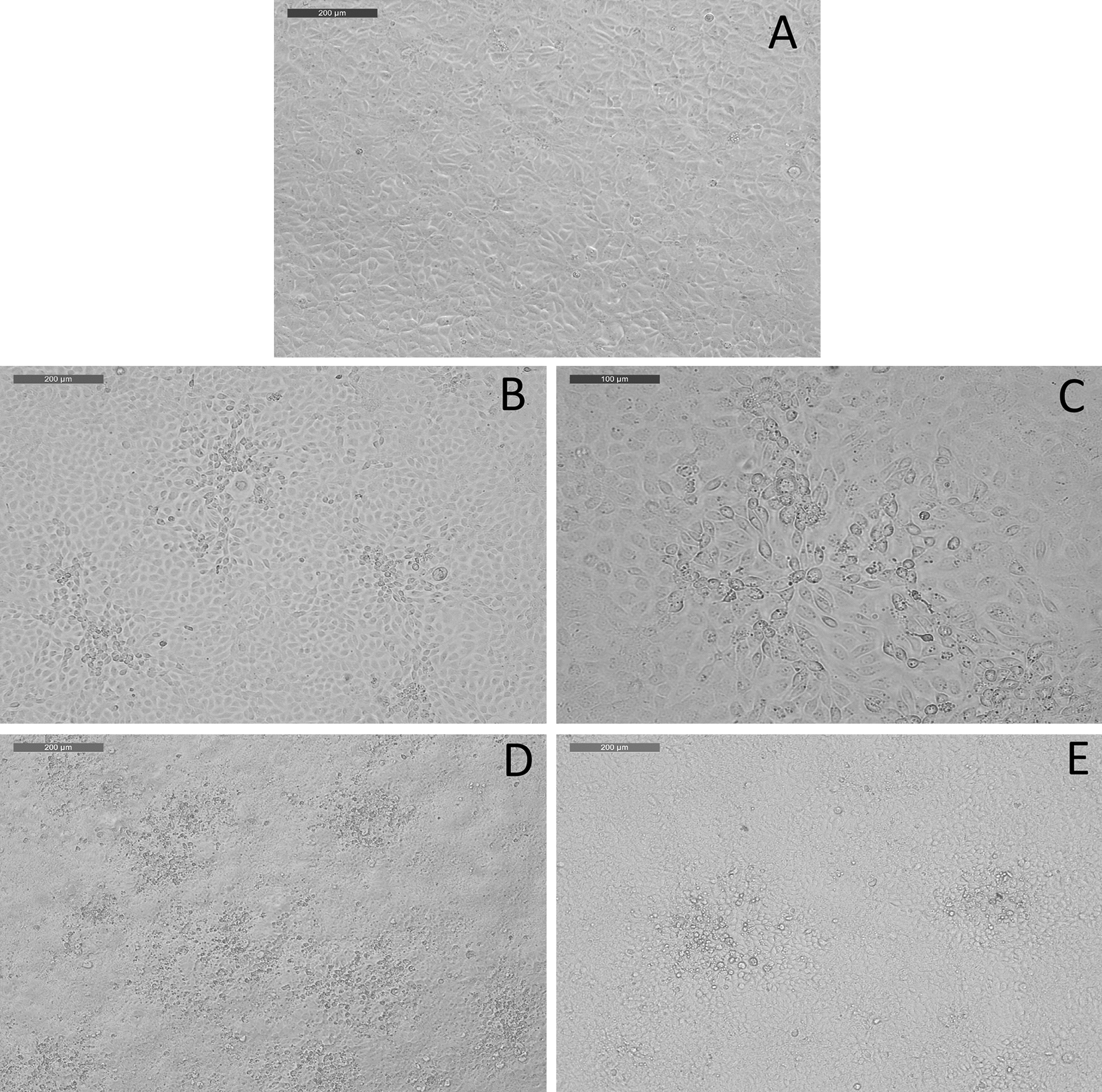
**Cytopathic effect observed on CHSE**_**214**_** cell line incubated with a diluted tissue homogenate from sample OO76.** **A** Non-infected cells; **B**, **C** Cells at day 6 post-infection; **D**, **E** Infected cells turn in round-shaped and refringent cells, forming plaques, at 8 dpi.

Extracts of internal organs and ascites from the abdomen were inoculated on five different cell lines. A slight CPE, characteristic of viral presence, was detected after 17 days of culture on the CHSE_214_ cell line incubated at 20 °C, and after one month at 14 °C for the internal organ sample. A second passage performed after 7 and 14 days of culture did not allow viral amplification on other cell lines. Infected CHSE_214_ cells appeared rounded and refringent, with aggregates sloughing from the cell monolayer and vacuoles (Figure 2). Cells located around the CPE appeared larger than those further away on the monolayer. No CPE was observed during the 21 days following contact between the first passage positive supernatant and chloroform. After several passages, the viral replication cycle seemed to be faster and more characteristic, with first CPE signs appearing within less than a week. Moreover, slight CPE on BF-2 cells inoculated with a positive supernatant from a first passage on CHSE_214_ was eventually obtained after three weeks of culture. The cultured virus was named OO76_s_.

**Figure 3:**
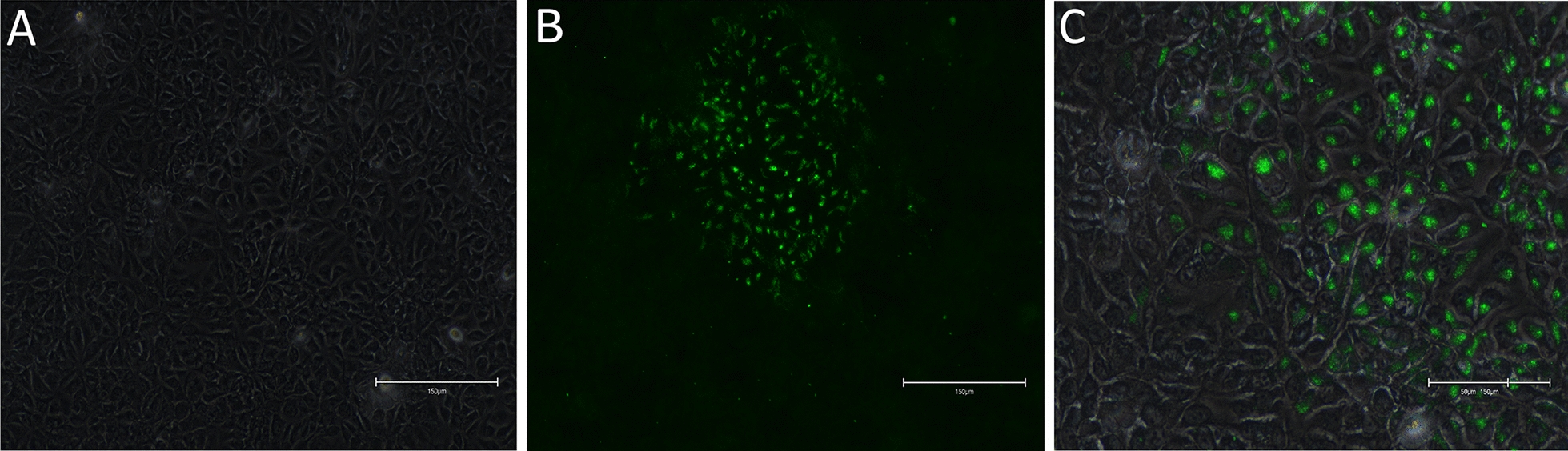
**IFAT detection of dsRNA in CHSE**_**214**_** cells infected with homogenate 16/219 using a J2 monoclonal antibody.** Infected cells are characterized by green, fluorescent cytoplasm. **A** Uninfected CHSE_214_ cells; **B** CPE observed with green fluorescent protein (GFP) filter (objective × 10); **C** Superposition of color (RGB) and GFP pictures (objective × 20).

Symptomatic fingerlings from another batch were delivered to the laboratory 4 months later (sample 16/219_p_), and homogenates from the internal organs and tissues from whitish three-dimensional lesions were inoculated on BF-2 and CHSE_214_ cell lines. CPE was observed only in CHSE_214_ cells inoculated with tissue homogenates from whitish three-dimensional lesions after 3 weeks at 20 °C, corresponding to a cultured virus named 16/219_s_. IFAT performed using the J2 antibody revealed dsRNA in OO76_s_- and 16/219_s_-infected cells, whereas no fluorescence was observed in the negative control (Figure 3). The fluorescence appeared to be localized exclusively in the cell cytoplasm and strictly matched the CPE area.

### Transmission electron microscopy

**Figure 4:**
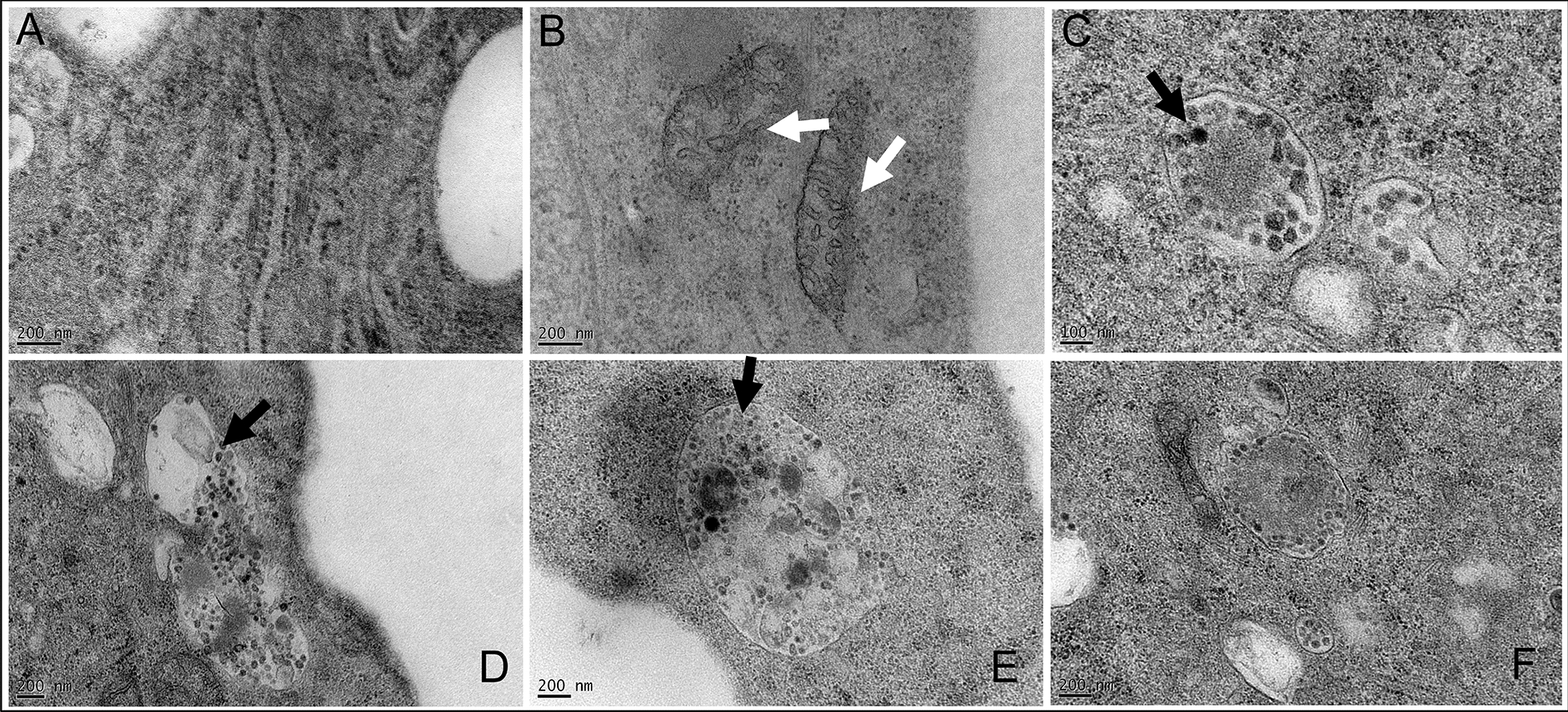
**TEM of CHSE**_**214**_** cells infected with a diluted tissue homogenate from sample OO76.** **A** Negative control; **B** to **F** Infected CHSE_214_ cells 7 dpi. Spherical particles vacuolized in endosomes are shown with black arrows. White arrows underline shrinkage of mitochondrial membrane.

Spherical particles of approximately 50 nm in diameter were detected by TEM in the cytoplasm of CHSE_214_ cell line positive for OO76_s_, especially at 5 dpi at 20 °C (Figure 4). They were also observed at 7 dpi, but in smaller quantities. A high degree of degradation of the mitochondrial envelope, which appeared to be shrinking, was also observed. No spherical particles were observed in the uninfected cells.

### High-throughput sequencing of the viral genomes and ORF organization

RNA extracted from the OO76_s_ CHSE_214_ cell supernatant was processed for high-throughput sequencing. The nearly 10 million reads obtained (average length 123 bp) were aligned with bowtie2-veryfast/megablast to a locally installed NCBI 'nt' database with an in-house script, allowing taxonomic assignment for rapid characterization of the reads from a blast tabular equivalent output. Most of the reads were of cellular origin and matched the Chinook salmon genome, but a few hundred reads (377) gave positive hits, with identities between 95 and 100%, to an NS5B pestivirus sequence.

Among the contigs generated after assembly, a contig of 13,123 nt was obtained. After manual curation of the alignment, we obtained a 387-nt 5' UTR, followed by a sequence coding for a polyprotein of 4,013 amino acids, and a 964-nt 3' UTR, relevant to a virus belonging to the *Flaviviridae* family. Characteristics of the N^pro^ sequence were observed in the proximal part of the genome, as well as the E^rns^ sequence, with an HEWQKHG motif matching RNase activity.

Interestingly, direct next-generation sequencing (NGS) analysis of tissue homogenates from whitish three-dimensional lesions of infected fingerlings (16/219_p_) confirmed the presence of the flavivirus, but also revealed the presence of a 5,068-nt long astro-like virus sequence. This sequence was partial and codes for 2 ORFs putatively characterized as the non-structural proteins ORF1a (partial) and ORF1b. The highest amino acid identities for ORF1b (RNA-dependent RNA polymerase) were observed with an astrovirus previously described [39] in common smelt (*Retropinna retropinna*) in New Zealand [36% identity over a length of 943 aa (WLJ60740.1)] and with *Hippocampus erectus* astro-like virus 1 [52% identity over a length of 438 aa (WEY37092.1)]. The presence of both viruses was confirmed using specific RT-qPCR, which displayed Ct values of 22.9 and 21.5 for flavivirus and astro-like viruses, respectively.

### Phylogenetic analyses

**Figure 5:**
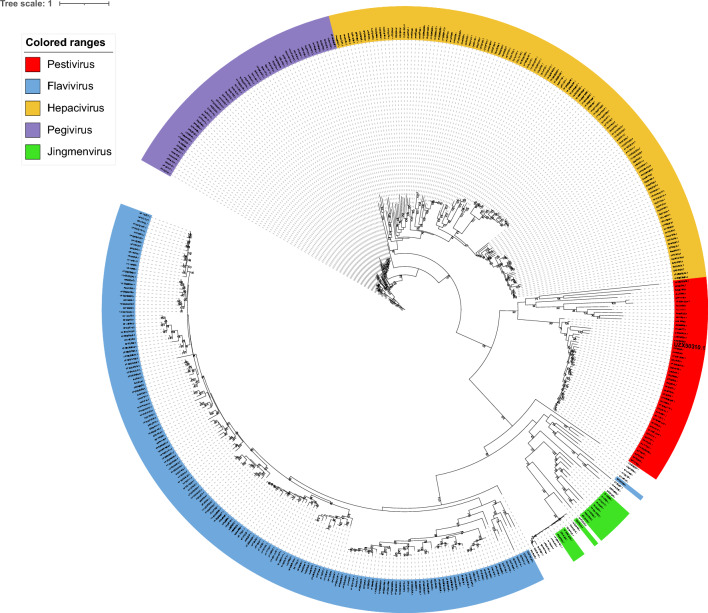
**Maximum likelihood (ML) phylogenetic tree representing a large panel of viruses belonging to the *Flaviviridae* family**. Using a phylogenetic tree based on 172 amino acids of NS5b with 428 sequences, the LG + F + I + R7 substitution model was selected and ultrafast bootstrap approximation (1000 replicates) was used. OO76_s_ seabass pesti-like virus (UZX50310) is represented in bold. The color strip highlights the various main genera described for Flaviviridae. Only the values for bootstrap support above 70% are represented.

**Figure 6:**
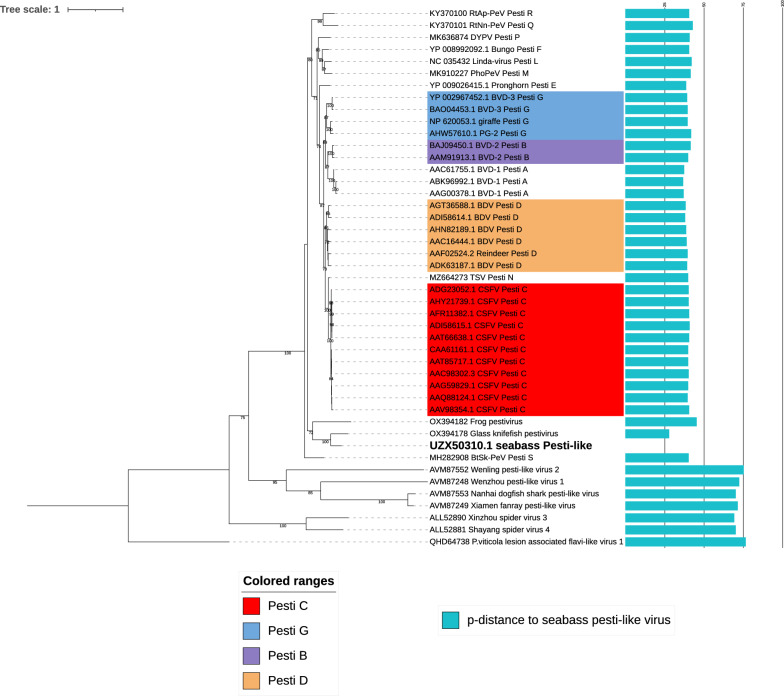
**Maximum likelihood (ML) phylogenetic tree focused on the *Pestivirus* genus members or putative members.** The tree was reconstructed based on the 314 amino acid sequences of partial NS5b from 45 viruses, with the LG + I + R4 substitution model and ultrafast bootstrap approximation (1000 replicates). The values for bootstrap support are represented by color codification, i.e., red for values from 0 to 15 and green for values from 78.75 to 100. P-distances between DLPLV and other *Pestivirus* members are represented by blue bars (scale from 0.1 to 0.4). DLPLV (UZX50310) is in bold.

A phylogenetic tree was reconstructed for the flavivirus based on the polyprotein sequence (Figure 5). Our *Dicentrarchus labrax* Pesti-like virus (DLPLV) OP499893 clustered with Pestiviruses and differed from viruses of genera such as Flaviviruses, Pegiviruses, and Hepaciviruses. Other flavi-like viruses detected on aquatic hosts clustered with either Hepaciviruses (Western African lungfish hepacivirus AVM87255; Xiamen guitarfish hepacivirus AVM87253; Nanhai ghost shark hepacivirus 2 AVM87258), or Flaviviruses (Southern pygmy squid flavivirus QCH00711; Lumpfish flavivirus YP009551951), except the Glass knifefish pestivirus OX394178 and the frog pestivirus OX394182. Regarding the phylogeny restricted to the NS5B protein and focused on *Pestivirus* genus (314 residues, Figure 6), it seems that the DLPLV UZX50310 clustered on a separate branch, not far from the glass knifefish pestivirus OX394178 and the frog pestivirus OX394182, which fell in a basal position. The newly described DLPLV displayed minimal and maximal p-distances of 0.27 with the glass knifefish pestivirus and 0.75 with the Wenling_pesti-like_virus AVM87552. In comparison, the p-distance within viruses from the species Pesti A ranged from 0.03 to 0.08, within viruses from the species Pesti D from 0.08 to 0.13, and a virus from the species Pesti Q displayed a p-distance of 0.38 with Pesti A representative viruses, and from 0.38 to 0.41 with Pesti D viruses (Figure 6).

**Figure 7:**
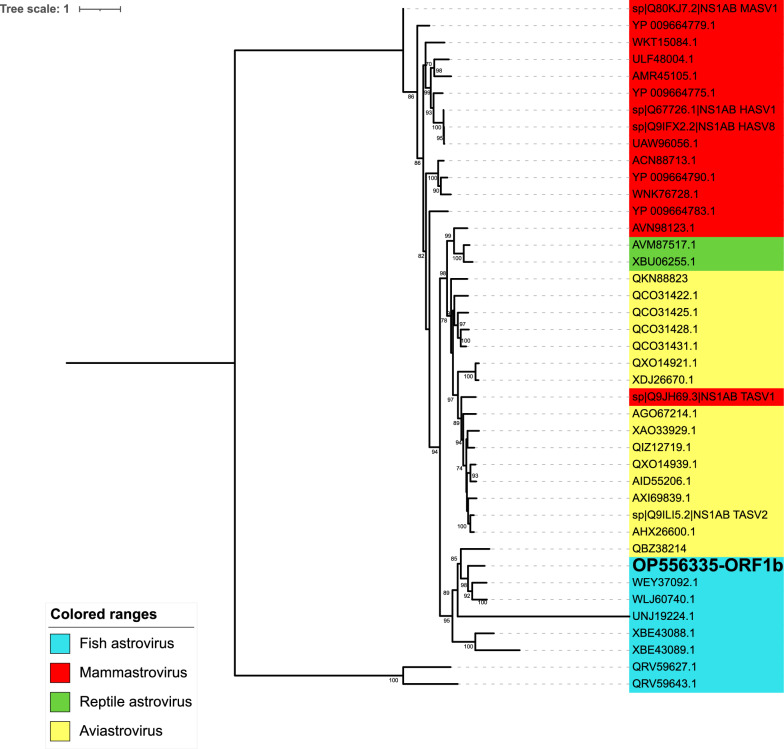
**Maximum likelihood (ML) phylogenetic tree built with *Astroviridae* family members**. The tree was reconstructed based on the 319 amino acid sequences of partial ORF1b from 41 viruses, with the LG + R5 substitution model and ultrafast bootstrap approximation (1000 replicates). The values for bootstrap support are represented in percent. *Dicentrarchus labrax* astro-like virus DLALV (OP556335) is in bold.

Regarding the astro-like virus, phylogenetic analysis of RdRp amino acid sequences (41 sequences, 319 residues) representing the two main subgroups, *Aviastrovirus* and *Mamastrovirus*, showed that the newly characterized *Dicentrarchus labrax* astro-like virus (DLALV) clustered with other fish astroviruses, such as Retropinna astrovirus 1 WLJ60740 (59.03% id), Eviota astrovirus XBE43089, and Toitoi astrovirus 1 UNJ19224 (Figure 7), but also with aviastrovirus European roller astrovirus QBZ38214, displaying an amino acid identity of 53.56%.

### Experimental trials

First, assays were performed to investigate the effects of simultaneous infection with DLPLV and DLALV and to assess routes of transmission. This was done by placing diseased fingerlings showing clinical signs (shedders; from a batch analyzed as positive for DLPLV and DLALV with Ct values of 14.9 and 29.4, respectively) either in direct contact with disease-free fish (cohabitants) or in water-only contact, using a basket placed inside the tank. All distended belly shedders progressively died within 16 days, and dead fish (n = 5) analyzed at 12 dpi revealed a positive signal using specific DLPLV and DLALV RT-qPCRs (Ct values 23.6 and 24.4, respectively). Whitish three-dimensional lesions were observed on approximately 25% of the cohabitants in water-only contact (positioned in the basket) at 23 dpi, but they rapidly disappeared after 2 or 3 days without triggering any mortality (Table 1). Specific RT-qPCR performed at 36 dpi on a pool of three fish from the basket revealed a negative signal for DLPLV (primer set oPVP645-646), whereas a positive signal was observed for DLALV PCR (primer set oPVP743-742, Ct = 28.0). Another PCR analysis was carried out at the end of the experiment, at 58 dpi, on 3 pools of 3 fish from the tank and basket. While only pools of fish from the tank yielded positive results for DLPLV (3/3, Ct ranging from 26.8 to 30.5), DLALV was detected only in pools from the basket (3/3, Ct ranging from 30.5 to 31.5, Table 1). No clinical signs or mortality were observed in cohabitants placed directly in contact with shedders, and survivors were positive only for DLPLV by cell culture and RT-qPCR, and not for DLALV.

**Table 1:** **Investigation of the route of diffusion of DLPLV and DLALV**

Condition	Infection	Mortality (%)	Dead fish			Survivors	
			CC	RT-PCR DLPLV	RT-qPCR DLALV	CC or RT-qPCR DLPLV	RT-qPCR DLALV
Fish with distended bellies	/	15/15 (100%)	** + **(n = 5)	** + **(n = 5)	+ (n = 5)	/	/
						**36 dpi**	
Fish from the basket	Water	0/20 (0%)	/	/		**–** (n = 3, 1 pool)	** + **(n = 3, 1 pool)
						**58 dpi**	
						– (n = 3, 3 pools)	+ (n = 3, 3 pools)
Fish from the tank	Direct contact	0/25 (0%)	/	/		** + **(n = 3, 3 pools)	**–** (n = 3, 3 pools)

**Figure 8:**
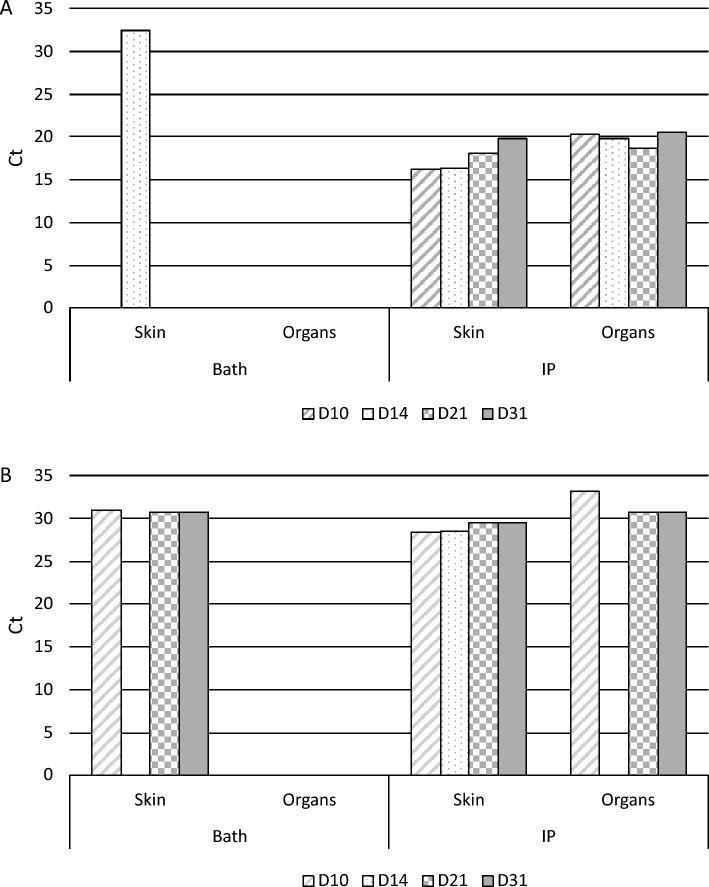
**Kinetics of DLPLV and DLALV loads in skin and organs of seabass fingerlings infected by bath or IP injection with 16/219p homogenate.** Specific RT-qPCRs were performed and Ct values are indicated at four times post-infection (10, 14, 21 and 31 days). For each date, a pool of 3 individuals per condition was analyzed. The absence of a bar means that the virus was not detected. **A** DLPLV viral loads; **B** DLALV viral loads.

The infectivity of 16/219_p_ on 51 dph fingerlings was assessed through bath or IP injection. No mortality or clinical symptoms were observed during the 1-month monitoring period, regardless of the infection mode. RT-qPCR analyses showed that DLPLV was present only sporadically in the skin after immersion (Ct = 32,4 at 14 dpi), while it was detected from 10 to 31 dpi after IP infection in the skin (Ct_mean_ = 17.6) and internal organs (Ct_mean_ = 19.8; Figure 8A). DLALV was repeatedly detected only on the skin (Ct ranging from 31 to 30.7) for the immersion condition, while it was detected on the skin (CT_mean_ = 29.0) and internal organs (Ct_mean_ = 31.5) after IP injection (Figure 8B).

In addition, to reproduce the clinical symptoms observed in the hatchery and to monitor the infection kinetics of the cultivable DLPLV, seabass fingerlings at two different ages were experimentally infected with OO76_s_ by bath or IP injection. At 162 dph, mortality was observed in fingerlings infected by bath at 25 to 50 dpi, with cumulative mortality reaching 8 and 16% for the two replicates (Table 2). Clinical changes were observed the day before death, including darkening of the skin, lethargy and a marked loss of appetite. At 26 and 31 dpi, dead fish (n = 2) were individually tested using cell culture and RT-c and -qPCRs specifically designed for the DLPLV (primer sets oPVP469-470 and oPVP645-646, respectively). A CPE was observed for each sample, as well as either PCR product at the expected size or specific amplification curve (melting temperature, Tm = 82.3 °C). Slight mortality (2%) was observed for non-infected control fish, without virus detection by cell culture or RT-PCR. Survivors, controlled by RT-cPCR and RT-qPCR at 53 dpi, exhibited DLPLV-positive signals (Table 2). The serum neutralization test, performed on plasma sampled at 81 dpi, was validated by the absence of specific antibodies in the negative control (Table 3). Antibodies specific to DLPLV were detected in 85% of the bath-infected fish and 100% of the IP-infected fish. A proportion of 80% of the seropositive immersed fish demonstrated high titers of at least 320. For the IP condition, 100% were higher than 320 (Table 3).

**Table 2:** **Experimental infection of seabass fingerlings with OO76**_**s**_** (DLPLV)**

Condition	Infection	Mortality (%)	Dead fish		Survivors
			CC	RT-PCR	CC or RT-PCR
Negative control	Bath	2/100 (2%)	–	–	–
Infected fish		4/50–8/50 (8–16%)	** + **(n = 2)	** + **(n = 2)	** + **(RT-PCR; n = 3)
Negative control	IP	0/50 (0%)	/	/	–
Infected fish					
		0/50 (0%)	/	/	** + **(RT-PCR, n = 3, 1 pool)

Dead fish were assessed individually by cell virology or specific RT-PCR after sampling skin and viscera. Three survivors/condition (either bath or IP, and uninfected or OO76_s_) were sampled (skin and viscera) at 53 dpi, and homogenates were further inoculated on cells or processed through RT-PCR targeting DLPLV.

**Table 3:** **Proportion of DLPLV neutralizing antibodies in infected seabass survivors at 81 dpi**

Expected serological status for OO76_s_	Sample identification	Number of samples	Percentage of DLPLV positive samples obtained using the SN test (n)	Titer in anti-DLPLV neutralizing antibody (min–max)
Negative	NEG1 to NEG20	20	0	/
Positive	OO76 bath1 to bath40	40	85 (34)	80– > 320
	OO76 IP1 to IP10	10	100 (10)	> 320

**Figure 9:**
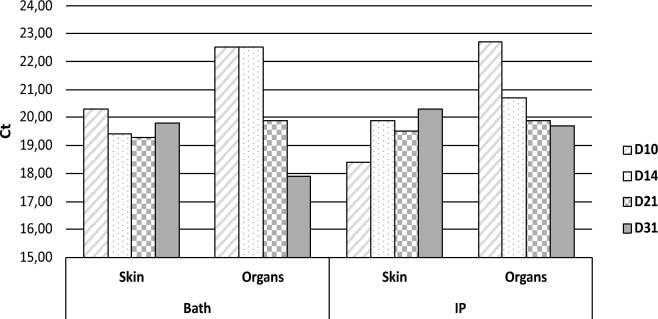
**Kinetics of DLPLV viral loads in skin and organs of seabass fingerlings infected by bath or IP injection with OO76**_**s**_** supernatant.** Specific RT-qPCRs were performed and Ct values are represented at four times post-infection (10, 14, 21 and 31 days). For each date, a pool of 3 individuals per condition was analyzed.

At an earlier physiological stage (51 dph), no clinical symptoms appeared during the month of monitoring, regardless of the route of inoculation (immersion, IP injection). DLPLV was detected at any time of sampling (10, 14, 21 and 31 dpi) on the skin as well as in internal organs (Figure 9), with Ct ranging for internal organs from 22.5 to 17.9 to and 22.7 to 19.7 to for the bath and IP infection routes, respectively.

## Discussion

This study aimed to characterize the putative causative agent of newly described disease affecting seabass fingerlings in a hatchery that causes whitish three-dimensional lesions on fins and sides, distended abdomens and low mortality. The combination between inoculation of different tissue homogenates on a large panel of fish cell lines and NGS, directly or after amplification in cell culture, led to the description of two viruses, one potentially belonging to the *Pestivirus* genus inside the *Flaviviridae* family, and other from the *Astroviridae* family.

An unknown virus was first isolated after a long cell cultivation period from infected fish organs and a similar CPE could be observed additionally after inoculation of a homogenate prepared from external skin three-dimensional lesions sampled on diseased fish. Positive IFAT labeling using J2 antibody confirmed the detection of double-stranded RNA, possibly generated during the replication of a positive sense genome virus, and the existence of a viral envelope was demonstrated through a chloroform test. NGS analysis of cell culture supernatant showed that OO76_s_ corresponds to an RNA virus with a genome of approximately 11 kb, coding for a large polyprotein of 40,13 amino acids, an organization compatible with members of the *Flaviviridae* family. Viruses belonging to the *Flaviviridae* family are usually described as enveloped viruses with a spherical shape, 50 nm in diameter, with non-segmented, single-stranded, linear and positive sense RNA genomes of about 9 to 13 kb [9]. Their genomes encode a polyprotein, later cleaved into several structural and nonstructural proteins. High genome diversity is described within the four genera characterized in this family. To date, the genus *Pestivirus* comprises nineteen species, defined by at least 25% differences in their coding nucleotide sequences [18]. The glycoprotein sequence of DLPLV, which looks divergent and longer than well-described pestiviruses such as Bovine viral diarrhea virus (BVDV), harbored two motifs corresponding to nuclease (E^rns^) and protease (N^pro^) activities specifically present in the envelope glycoprotein of pestiviruses. The amino-acid sequence HEWQKHG, and particularly its histidine component involved in the RNase activity of the E^rns^ motif, was identified on the DLPLV sequence. This nuclease has the capacity to cleave single-stranded and double-stranded viral RNA, preventing the stimulation of the host’s interferon (IFN) response [21] and participating in the establishment of persistent infections. Similarly, the N^pro^ motif is involved in the suppression of IFN I pathway antiviral response [22]. Although these motifs appear to be almost systematically conserved among pestiviruses, they could not be demonstrated in certain species such PhoPeV, a novel pestivirus described on porpoises [40]. Virus-like spherical particles of approximately 50 nm were observed by TEM, with numerous particles clustered in endosomes. These observations are consistent with viruses belonging to the *Flaviviridae* family, which are known to acquire their envelope through budding from the endoplasmic reticulum, then moving in cells thanks to intracytoplasmic vesicles [41]. To date, Flaviviruses, usually arthropod-borne viruses, have been described in a large range of hosts, but were believed to infect mostly humans and mammals. In recent years, there has been some evidence of other potentially susceptible animals, such as rats [42], bats [43] or even nematodes [44]. Moreover, the increasing number of deep sequencing analyses in the aquatic animal field, particularly in teleost fish, led to the discovery of numerous unknown viruses, among which some were closely related to the genus *Flavivirus*. In 2016, a new virus, named the Wenling Shark Virus (WLSV), belonging to the genus *Hepacivirus*, was described after transcriptome analysis on tissue from a catshark *Proscyllium habereri* [13]. A few years later, two other putative flaviviruses were reported on lumpfish [12] and pacific salmon [15]. Considering these recent descriptions, Postel et al*.* adapted the classification within the genus *Pestivirus* by including eight additional species to the eleven already described based on the identified or putative hosts [17, 18].

The second viral agent, an astro-like virus named DLALV, was detected by deep sequencing in diseased fish showing whitish three-dimensional skin symptoms. Astroviruses are single-stranded, positive-sense, 28–30 nm in diameter, RNA viruses that have long been isolated from stools of a wide range of mammals and birds, mostly associated with gastroenteritis in young individuals [45, 46]. Recent discoveries of new astroviruses or astro-like viruses suggest, however, broader tropism of this viral family which appears to also be prevalent in aquatic environments, as suggested by Geoghegan et al*.* who reported that 39% of all the viruses discovered in seven marine fish species could be astroviruses regarding transcript abundancy [47].

Experimental infections were performed to study the replication kinetics and the potential pathogenicity of DLPLV and DLALV. Seabass fingerlings (162 dph) were infected with a cell supernatant of the DLPLV via bath or intraperitoneal injection. No clinical signs were reported, but low mortality was observed (8–16%) by bath, with re-isolation of virus on cell culture and PCR detection of the virus from dead fish as well as from survivors at 53 dpi. This observation suggests that DLPLV could pass the natural physical barrier and persist in infected specimens for almost two months. In younger fish (51 dph), no mortality was observed despite the virus being detected at high levels in the skin and organs from 10 to 31 dpi, regardless of the route of infection, with a slight increase in viral load in organs over this period of time (Ct ranging from 22.50 to 17.90 and 22.70 to 19.70 for bath or IP, respectively). It would be interesting to supplement this data with earlier post-infection analyses, which would allow for more precise determination of the viral load kinetics.

In the cohabitation assay, where shedders with symptomatic signs were positioned in direct or indirect contact with naïve cohabitants, typical clinical signs such as whitish three-dimensional skin lesions were observed very fleetingly and only on fish in the basket. Surprisingly, DLPLV was detected only in cohabitants from the tank and not in the basket, suggesting the absence of water-borne transmission. DLALV was detected on several pools of survivors from the basket at different times post-infection and could be the etiological agent responsible for the skin symptoms. Surprisingly, it seems that DLALV was not detected in cohabitants directly in contact with shedders, at 58 dpi. As no sampling was performed earlier (only cohabitants from the basket were sampled at 36 dpi), it could be hypothesized that DLALV was cleared from cohabitants before 58 dpi. Regarding the trial with tissue homogenates from whitish three-dimensional skin lesions positive for both viruses, DLPLV was only detected in one skin sample with a weak signal in the immersion condition, while it was detected with earlier Ct in fish infected by IP injection, suggesting that the virus may encounter some difficulties to efficiently infect fish through natural ways (skin, gills) but would be able to persist in fish once these physical barriers are passed. This apparent absence of virus may indicate a lack of infectiousness through the natural route of infection (entry into the host via skin or gills), except if the virus has been amplified in vitro before. These observations could be partially explained by the existence of two biotypes in cell cultures, as already described for most pestiviruses, corresponding to a cytopathic population (cp) responsible for cell death, and a noncytopathic (ncp) one, able to replicate at low rates, without triggering severe damage. These two biotypes may be correlated with the transient or persistent character of the infection [48]. Bovine viral diarrhea virus (BVDV), for instance, is responsible for asymptomatic infections but can also lead to death through the development of mucosal infection [49]. The in vitro multiplication of DLPLV made possible the design of a seroneutralization test. Analyses of sera sampled from infected fish at 81 dpi demonstrated the presence of specific antibodies, regardless of the route of infection (immersion or IP injection). Thus, even though no clear clinical signs were observed on infected fish, DLPLV could enter the host and trigger adaptative and specific immune response, leading to specific neutralizing antibody production. The apparent stable viral load in tissues, skin or internal organs, suggests viral persistence and possible equilibrium between viral replication and host immune response. In calves, differences in levels of serum neutralizing antibodies were observed between ncp and cp BVDV, suggesting that ncp strains induced a stronger immune response than cp ones [50].

DLALV was detected in diseased fish showing whitish three-dimensional skin symptoms, and, in the cohabitation trial, cohabitants from the basket revealed positive signals for this virus, suggesting water-borne transmission. Moreover, only skin samples from fish infected by immersion displayed positive signals. Nevertheless, as skin and internal organs were not processed separately in the cohabitation assay, it remains difficult to strictly compare the two sets of results and to be sure that the virus was able to pass through the natural barrier of the skin and was not only detected on the external surface of fish. This virus seems to be able to persist for 1 month in infected fish, but at a weak level, even though the entry barrier is bypassed through IP injection. Unfortunately, these experiments were not designed to provide information on tissue tropism in seabass. Astroviruses were formerly described as viruses targeting the gastrointestinal tract [45], whereas recent discoveries have tended to enlarge this tropism to the brain [51] or respiratory tract. Regarding the clinical symptoms described here, distended bellies could be a consequence of gastrointestinal tract failure associated with inflammatory response and ascites production.

Considering the results of in vivo studies, one hypothesis is that the clinical signs observed in hatcheries could be multifactorial in origin, as observed for the Pacific oyster mortality syndrome [52]. Bacteria such as *A. fischeri* or *Tenacibaculum* spp. have been detected in some samples from symptomatic fish. Certain strains of *A. fischeri* have been reported to be pathogenic to *Scophthalmus rhombus* [53], and *Tenacibaculum maritimum* is well known to induce ulcerative disease, causing high mortality in various marine fish species worldwide [54]. Although hatcheries are well-controlled environments, the microbial diversity present in pre-growing tanks can clearly contribute to creating conditions favorable to the emergence of disease.

**Table 4:** **List of GenBank accession numbers, Bioprojects, Biosamples and SRA for the two submitted sequences (SeqId)**

Accession number	SeqId	Bioproject	Biosample	SRA (SRR accession)
OP499893	Pestivirus isolate OO76_s_	PRJNA120777	SAMN46143980	SRR35708613
OP556335	Astro-like_virus_ Dicentrarchus labrax virus isolate 16–219_p_ ORF1a gene, partial cds; and ORF1b gene, complete cds	PRJNA120777	SAMN46143981	SRR35708612

In this work, the combined use of different diagnostic tools made it possible to characterize two new viruses, DLPLV and DLAVL, in seabass fingerlings showing whitish three-dimensional lesions and distended bellies in a hatchery. This clinical episode was fleeting and induced only low mortality; however, it generated significant economic losses. No trace of the two characterized viruses could be detected after disinfection of the affected tanks, and no new cases have been reported since, in this or other hatcheries. Even though the potential involvement of one or both viruses in the observed clinical signs cannot be formally proven based on the experimental trials conducted, it is important to remain vigilant in a context of global climate change, which could have an impact on the biology of these agents. To this end, the diagnostic tools developed will enable specific epidemiological surveillance in farmed and wild fish.

## Supplementary Information

**Additional file 1. Details on experimental trials:** objectives, experimental conditions, fish development stage, inoculum viral load, and sampling time.

## Data Availability

The virus genome sequences in this study are available in GenBank under the following nucleotide accession numbers: OP499893 for OO76s-1st passage on CHSE214 and OP556335 for 16/219p. Sequencing data were submitted to the NCBI Sequence Read Archive and are available under the BioProject accession numbers listed in Table 4.
