# Supplementary material for: Characterization of novel pesti- and astro-like viruses in hatchery-reared European seabass (Dicentrarchus labrax) associated with health disorders and low mortality
Source: Vet Res. 2026 May 19;57:79. doi: 10.1186/s13567-026-01758-2 (PMC13188376; doi:10.1186/s13567-026-01758-2)
Supplement: Supplementary file 1 — Additional file 1. Details on experimental trials: objectives, experimental conditions, fish development stage, inoculum viral load, and sampling time. [file 13567_2026_1758_MOESM1_ESM.docx]

**Additional file 1.** Details on experimental trials: objectives, experimental conditions, fish development stage, inoculum viral load, and sampling time.

|  |  |  |  |  |  |  |
| --- | --- | --- | --- | --- | --- | --- |
| Objective | Condition | Age (dph) | Inoculum (infectious titer and/or Ct) | |  | Sampling (day post-infection or contact) |
|  |  |  | Pestivirus | Astrovirus |  |  |
| *Effect of simultaneous infection with pesti- and astro-viruses* | 25 disease-free fish in direct contact with 15 shedders (distended bellies) + 20 disease-free fish in a basket | 162 | Viral load obtained in 5 shedders : | |  |  |
|  |  |  | Ct 14.9 | Ct 29.4 |  | 36, 58 |
|  | 40 disease-free fish infected by bath or intraperitoneal (IP) injection with 16/219p | 51 | Ct 22.9 | Ct 21.5 |  | 10, 14, 21, 31 |
| *Virulence and infection kinetics of pestivirus* | 300 disease-free fish infected by bath or IP injected with 0076s | 162 | 1.5E+7 TCID_50_ in 3L of water (bath)  2.37E+4 TCID_50_ (IP) | - |  | 53, 81 |
|  | 40 disease-free fish infected by bath or IP injected with 0076s | 51 | Ct 12.5: 5 mL in 200 mL water (bath) 50µL injected / fish (IP) | - |  | 10, 14, 21, 31 |
